# Should cancer pain still be considered a separate category alongside acute pain and chronic non-cancer pain? Reflections on ICD-11

**DOI:** 10.3389/fpain.2024.1397413

**Published:** 2024-05-02

**Authors:** Emmanuel Bäckryd

**Affiliations:** Pain and Rehabilitation Center, and Department of Health, Medicine and Caring Sciences, Linköping University, Linköping, Sweden

**Keywords:** acute, cancer, chronic, classification, death, ICD-11, pain, taxonomy

## Abstract

**Introduction:**

Traditionally, cancer pain has often been viewed as an independent third major category in pain medicine alongside acute pain and chronic non-cancer pain. However, the new chronic pain category MG30 in the eleventh version of International Classification of Diseases (ICD-11) includes cancer-related pain as one of its seven subgroups. In light of this, the aim of the paper is to investigate whether the traditional trichotomy should be replaced by a dichotomy between acute pain and chronic pain, cancer-related pain being part of both groups depending on the duration of pain.

**Methods:**

The rationale for viewing cancer pain as a separate category is reviewed.

**Results:**

Cancer being a deadly disease, cancer pain has a life-and-death and existential dimension that is different from non-cancer pain. It seems sensible to believe that this is an additional dimension to the suffering caused by cancer pain, and that clinicians should therefore take this existential dimension into consideration when assessing pain.

**Conclusion:**

Without challenging the place of chronic cancer-related pain under the MG30 heading, it is concluded that while using ICD-11 in the future, pain clinicians should continue being mindful of the fact that the reality of death shapes the experience of cancer pain. The traditional trichotomy is therefore still valid and mirrors the fact that human beings are *vulnerable* (acute pain), *temporal* (chronic pain) and *mortal* (cancer pain).

## Introduction

### The WHO analgesic ladder

When *Lancet* in 2011 wanted to “give an overview of the latest developments in pain management” (1), they published three reviews covering the three main areas of pain medicine: acute pain, chronic pain, and cancer pain (2–4). Hence, traditionally, cancer pain has often been viewed as an independent third major category in pain medicine. One illustration of this pertains to treatment strategies. In 1986, the World Health Organization (WHO) published the three-step analgesic ladder (5, 6). The outspoken aim was freedom from *cancer* pain. The ladder was part of a health program aimed at improving strategies for cancer pain management through educational campaigns, the creation of shared strategies, and the development of a global network of support (7).

The WHO analgesic ladder can be viewed as an important step away from the period of opiophobia inaugurated by the Poisons and Pharmacy Act in Great Britain in 1908 and the Harrison Act in the USA in 1914 (8). Of course, the opioid epidemic that was unleashed in the USA around the turn of the millennium signified the death of opiophobia and the victory of what has been termed opiophilia (9) or opiocentrism (10). In many parts of the world however, opiophobia still prevails, leading to a strange paradox: in some parts of the world, people die because of opioid overprescribing; in other parts of the world people die in needless pain because of the lack of basic opioid-based pain relief. All in all, cancer pain as an independent pain category makes a lot of sense when viewed from historical and pharmacological perspectives: in the battle against opiophobia, this huge group of patients was identified as particularly suitable for treatment with opioids.

### Cancer as a chronic disease and the challenge of ICD-11

In 1996, WHO wrote that, for cancer patients having advanced disease, “the only realistic treatment option is pain relief and palliative care” (5)^p.v^. Having advanced cancer was in many ways more or less synonymous with end-of-life care, and therefore the word “chronic” applied best to non-cancer patients. Hence, it made sense to view cancer pain as a major category within pain medicine; cancer pain was seen as radically different from both acute pain and chronic non-cancer pain. However, a quarter of century later, oncology has made tremendous progress, and advanced cancer is no longer synonymous with imminent end-of-life care. The concept of *cancer survivors* is crucial here, and it is important to understand that there is an overall “blurring [of] previous lines of distinction in treatment strategies”, not least as “cancer evolves into a chronic illness” (11).

The 5-year-survival after cancer now exceeding two thirds, how to assess pain in cancer survivors in general and the indication for opioid use in particular, is a very important question (12). However, it is important to acknowledge that the category of cancer survivors is in itself a heterogenous category, including both patients who after treatment are free of their cancer (but who may suffer from chronic pain related to the cancer treatment they have undergone) and patients who live with cancer as a chronic illness (12). Hence, pain mechanisms might differ widely within the group of cancer survivors.

The previously mentioned “blurring [of] previous lines of distinction in treatment strategies” is made explicit in the eleventh version of International Classification of Diseases (ICD-11), where cancer-related pain is one of seven subgroups under the heading “MG30 Chronic pain”. Hence, it could be said that ICD-11 challenges the traditional trichotomy. Should we perhaps only talk about a dichotomy of acute vs. chronic pain, cancer patients being part of both groups depending on the duration of pain?

## Results

### Is there anything special about cancer pain?

First, it is important to acknowledge that a distinction is often made between *cancer pain* and *cancer-related pain.* Simply put, cancer-related pain is the broader category of the two, encompassing on the one hand cancer pain proper (i.e., pain caused directly by the tumour) and, on the other hand, pain related to the *treatment* of cancer (i.e., pain being a side effect of chemotherapy, radiotherapy or surgery). This distinction is visible in ICD-11, the chronic cancer-related pain category (MG30.1) being subdivided into chronic cancer pain (MG30.10) and chronic post cancer treatment pain (MG30.11).

Without challenging the place of chronic cancer-related pain under the MG30 heading, I would nonetheless argue that it is still philosophically and experientially relevant to view cancer pain as a major category alongside acute pain and chronic non-cancer pain, and that it is important to take this into consideration when assessing pain. The reason for this is rather simple. Even though they are highly debilitating and suffering-laden, chronic *non-cancer* pain conditions such as for instance fibromyalgia or chronic so-called unspecific low back pain are not deadly diseases. Cancers are. Hence, cancer pain has a dimension that chronic non-cancer pain conditions lack—an existential life-and-death dimension.

### Cancer pain as reminder of our mortality

As the organism cannot afford not recognizing tissue damage, pain has survival value (13). Among sensory receptors, nociceptors have the peculiarity of being *nonadaptive*, i.e., nociceptors “continue responding (and thus sending action potentials) as long as the stimulus continues” (14). For pain, there is even a tendency for the *opposite* of adaptation—the phenomenon known as sensitization (15). Our nervous system is “wired” in such a way that we cannot ignore pain. This means that when we experience cancer pain, we cannot distance ourselves from being reminded of death. Arguably, this adds to the suffering experienced by the cancer patient in pain. It is not only that he/she experiences pain, a cancer patient also has to cope with the fact that what causes the pain is a deadly disease.

Notwithstanding the traditional claim that in about 90% of cases adequate analgesia can be achieved by following the WHO analgesic ladder (6, 16), the treatment of cancer pain remains suboptimal (17). Notably, up to approximately two out of three patients with advanced cancer report pain (18, 19). The phenomenon of breakthrough cancer pain must also be mentioned in this context. More than 30 years ago, breakthrough pain was defined by Portenoy & Hagen as a transitory exacerbation of pain that occurs on a background of otherwise stable pain in a patient receiving chronic opioid therapy (20). Often, breakthrough pain is categorized into spontaneous, end-of-dose failure, or incident pain (21). Breakthrough pain is common in cancer (22), and regardless of its cause, it seems sensible to postulate that each occurrence is a reminder for the patient that he/she carries a potentially deadly disease.

When humans experience cancer pain, they are therefore mercilessly and disturbingly reminded of their mortality. Cancer pain reminds us of death because it is caused by a deadly disease, and it is merciless because we do not adapt to pain. Cancer pain is a powerful reminder of our existential situation—i.e., cancer pain is a form of pain that reminds us of the fact that we are mortal. There is a life-and-death dimension inherent to cancer pain which does not exist in acute pain or in chronic non-cancer pain.

### Cancer-related pain and ICD-11

ICD-11 works *de facto* with a dichotomous view of pain, acute pain being contrasted to chronic pain, and the latter explicitly encompassing chronic cancer-related pain as one of seven subgroups. A dichotomous rather than trichotomous view of pain is in many ways sensible and the present paper does not propose an alternative classification scheme. However, when assessing a pain patient, I submit that there are still good medico-philosophical and experiential reasons to continue view cancer pain as a separate category. It seems sensible to believe that there is an additional dimension to the suffering caused by cancer pain, and that clinicians should take that dimension into consideration when assessing a cancer pain patient. Of course, this also applies to other potentially deadly diseases that are painful. Perhaps one should talk about “deadly disease pain” rather than “cancer pain”. In other words, “cancer” is here used as a convenient shorthand for a disease that is life-threatening. All in all, while using ICD-11 in the future, clinicians should continue being mindful of the fact that the reality of death shapes the experience of cancer pain.

## Discussion

### Acute pain and chronic non-cancer pain

In two previous papers (13, 23), I have explored the experience of pain at the interface between clinical pain medicine and the philosophy of medicine. In the first paper, in dialogue with pain philosopher Murat Aydede, I investigated the concept of pain being not the experience of some-*thing* but of some-*one*, namely, the experience of the body that is I (13). This analysis of pain is congruent with the medical concept of acute pain, i.e., of pain as a symptom of potential or actual tissue damage. When I feel acute pain because of tissue damage, the pain tells me something about me and my place in this world, i.e., about how the body that is I is affected by the world. If I feel that the edge of a knife is sharp, I get epistemic access to the world outside me. But if the knife cuts through my skin, the pain that I will feel is not about the world outside me, it is about me and how the world affects me.

Chronic non-cancer pain is traditionally viewed as a second major area of pain medicine. In chronic pain, the pain is often not (merely) a symptom of something else but rather has turned into a disease in its own right. Simply put, chronic pain means that the pain system itself is “diseased”. As Melzack & Katz put it, chronic pain is “the result of neural mechanisms gone awry” (24). For obvious reasons, temporality is inherent in the concept of chronic pain, and, in a second paper (23), I therefore analysed the pain experience from the point of view of time. In dialogue with philosopher Fredrik Svenaeus, I argued among other things that pain chronification is a process in which the pain patient becomes aware of his/her temporality, both the past and the future coming to the fore. This contrasts with severe acute pain in which only the present counts, i.e., what counts is getting rid of the pain *now*, the patient being so to speak locked into the present (at least when the intensity of acute pain is very high). Hence, philosophically speaking, chronic pain makes us aware of our temporality, of the fact that we are temporal beings living in our own story. The medical burden of chronic pain, and the fact that it often can be viewed more as a disease than a mere symptom, is now recognized in ICD-11.

### The peculiarity of cancer pain

In the present paper, I have argued that despite the inclusion of chronic cancer-related pain in the MG30 category of ICD-11, cancer pain should nonetheless still be viewed as a third major category alongside acute pain and chronic non-cancer pain. However, some pain researchers disagree. Dennis C Turk, for instance, gives a number of reasons for a dichotomous (acute vs. chronic) instead of a trichotomous view (25):

• Using the terms malignant (i.e., cancer-related) vs. benign pain, Turk states that “to pain sufferers, however, no pain is benign”, implying that a trichotomous view would somehow lead to cancer-related pain being viewed as more important.
• The mechanisms of nociception being the same regardless of aetiology,”it makes no sense to discriminate between […] cancer and noncancer nociception”, according to Turk.
• Pain alters the nervous system whether it is associated with cancer or not.
• According to Turk, there is no evidence that pain-related emotions like anxiety or depressive feelings are unique to cancer pain.

From a pure biomedical perspective, the arguments made by Turk are essentially valid and uncontroversial. But pain is not only biology. Pain is a biopsychosocial phenomenon which, traditionally, is said to have three aspects: a sensory-discriminative, an affective-motivational (ie, pain as a “feeling”), and a cognitive-evaluative. And it is this last aspect which, I think, in part justifies a trichotomous view. The presence of a deadly disease is arguably not a little detail when a person with cancer experiences pain and thinks about its significance. In fact, Turk himself acknowledges this. In the same editorial, he writes (italics added):

I do not mean to suggest that neoplastic diseases have no unique qualities. They differ in *meaning*, in co-occurring noxious symptoms, and in the progression from many of the most prevalent chronic pain syndromes, but, I submit, the underlying nociceptive mechanisms are the same whether we are speaking of back pain, headache, rheumatoid arthritis, or metastatic cancer.

Importantly, Turk emphasizes “nociceptive mechanisms”, i.e., he gives biological reasons for his dichotomous view of pain. My contention is that this is too narrow a view. If, on the other hand, pain is viewed as a biopsychosocial phenomenon in which cognitive-evaluative aspects are important, then I think there are good reasons for considering cancer pain as a third major category. Moreover chronic non-cancer pains do not tend to respond well to opioids (26), whereas cancer pains often do. Hence, we come back to the WHO analgesic ladder and its advocacy for the judicious use of opioids against cancer pain. Also, in light of the devastating effects of the US opioid epidemic, I contend that the distinction between cancer pain and non-cancer chronic pain is still of value, e.g., when pondering whether to prescribe opioids or not. All in all, even though the lines are “blurred”, they have not dissolved into nothingness. They are still there to be seen.

**Figure 1:**
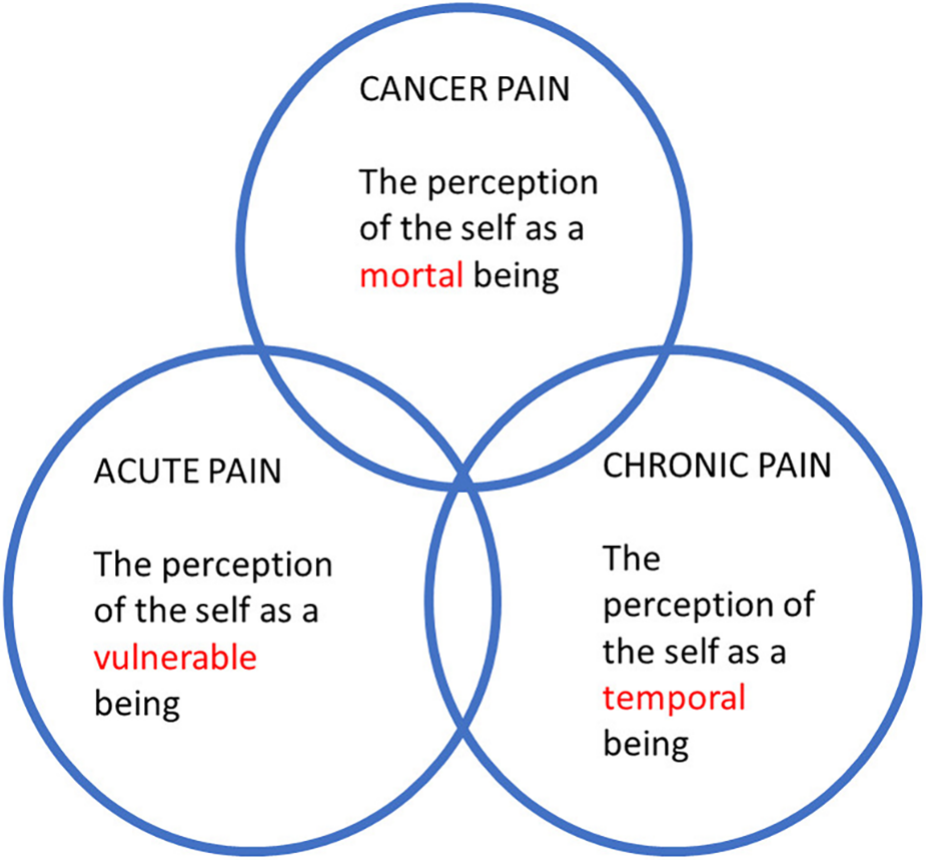
The trichotomy of acute, chronic non-cancer, and cancer pain from a philosophy-of-medicine perspective.

My three papers [the two previous ones (13, 23) and the present one] correspond to these three traditional areas in pain medicine, and there is an important progression involved in the publication sequence of the three papers. In short, I have tried to show that pain is the perception of the self as a *vulnerable*, *temporal* and *mortal* being (Figure 1). Let us unpack this, starting with “vulnerable”. When tissue damage occurs, *acute pain* reminds us of our vulnerability (13). The world is indeed a dangerous place, and acute pain can be seen as the perception of the self when the world “out there” has breached the physical integrity of the organism. Now to “temporal”. As was discussed in the second paper, pain can get chronic (23). For instance, a surgical wound might have healed perfectly well but a chronic pain has nonetheless developed. In such cases, the pain is not unlike a memory. Chronic pain is the result of “neural mechanisms gone awry” (24). I argued that *chronic pain* reminds us of our temporality and of the narrative character of our lives. And as the story inexorably ends in death, there is a clear link between “temporal” and “mortal”—which has been the subject of the present paper on *cancer pain*, with a focus on ICD-11. Hence, a coherent picture emerges in which the phenomenon of pain can be understood as a witness to human vulnerability, temporality, and mortality (Figure 1). The classical trichotomy seems to be very relevant from a philosophy-of-medicine and experiential point of view, and this should be weighed in when assessing pain.

### A short case study as an illustration

A 64-year-old man with an apical lung cancer on the left side suffers from an intractable neuropathic pain because of tumor infiltration in the brachial plexus. Due to metastases, he has other pain locations too and has been on long-acting morphine for months, but the pain radiating to the left arm has now become excruciating. An intrathecal catheter is introduced and, using fluoroscopy, the tip of the catheter is placed at the appropriate cervical segmental level. An intrathecal infusion of the local anesthetic bupivacaine is started, with an immediate analgesic effect. The patient is still not completely pain free (there are pains in other locations too), but the situation is nonetheless dramatically better. However, existential questions now become paramount for the patient. He describes it as follows: “Before, when the pain was so severe, like fire in the arm, I could not think about anything else but the pain. It’s great the pain is better, but now it is as if I have the strength to think about death. And that, well, it troubles in a different way.”

The case illustrates how a pain-related suffering can be “replaced” by existential suffering. The mild chronic pain the patient still experienced after the intervention (not least from other locations due to metastases) was not in itself a big problem; the problem now was that it reminded the patient of death and of his existential situation. [For a description of intrathecal analgesia, see e.g., the paper by Bäckryd & Larsson (27)].

## Conclusion

Different kinds of pain remind us of different aspects of our common human condition. For pain clinicians, such a philosophical understanding of pain is arguably an important aspect of empathy and respect for the suffering patient sitting or lying in front of us during a consultation. The fact that ICD-11 includes cancer-related pain as part of MG30 is not in itself a problem, provided that pain clinicians continue being mindful of the peculiarity of cancer pain. In other words, it is my contention that one can and should embrace the MG30.1 subsection of ICD-11 while at the same time conceptually holding on to the traditional trichotomy of acute pain, chronic non-cancer pain, and cancer pain.

## Data Availability

The original contributions presented in the study are included in the article/Supplementary Material, further inquiries can be directed to the corresponding author.
